# Source apportionment of soil heavy metals with PMF model and Pb isotopes in an intermountain basin of Tianshan Mountains, China

**DOI:** 10.1038/s41598-022-24064-1

**Published:** 2022-11-12

**Authors:** Tao Zeng, Long Ma, Yizhen Li, Jilili Abuduwaili, Wen Liu, Sen Feng

**Affiliations:** 1 State Key Laboratory of Desert and Oasis Ecology, Xinjiang Institute of Ecology and Geography, Chinese Academy of Sciences, Ürümqi, 830011 China; 2 Research Center for Ecology and Environment of Central Asia, Chinese Academy of Sciences, Ürümqi, 830011 China; 3 University of Chinese Academy of Sciences, Beijing, 100049 China

**Keywords:** Ecology, Environmental sciences

## Abstract

A boom in tourism may lead to the enrichment in heavy metals (HMs) in soils. Contamination with HMs poses a significant threat to the security of the soil environment. In this study, topsoil samples were collected from a tourist area of Sayram Lake, and the concentrations of HMs (Cr, Cu, Ni, Pb, Zn and Cd) were determined. With contamination and eco-risk assessment models, correlation analysis, Pb isotope ratios, redundancy analysis and positive matrix factorization (PMF) model, the risks and sources of HMs in the soil were studied. The *I*_*geo*_ results suggested that Cd was the primary pollutant in the tourist area of Sayram Lake. The potential ecological risk index (PERI) showed that the study area was at low risk, and the pollution load index (PLI) indicated that the study area had a moderate contamination level. Qualitative and quantitative analyses apportioned three sources of HMs, namely, natural sources (38.5%), traffic sources (27.2%) and mixed sources (tourist waste and atmospheric deposition) (34.3%). Redundancy analysis results showed that the HMs content was related to SiO_2_, Al_2_O_3_, TiO_2_, P_2_O_5_, MnO, K_2_O, Fe_2_O_3_ and SOC, and heavy metals tended to be stored in soil particles of grain sizes < 32 µm. These findings are expected to provide useful insights into the source identification of HMs in the soils of mountain tourism areas and provide a scientific decision-making basis for sustainable tourism development and for the assessment of ecological service values in the Tianshan Mountains.

## Introduction

Over the past decades, the impact of human activities such as the exploitation of mineral resources^1^, agricultural irrigation^2^, industrial development^3^, tourism and urban infrastructure^4,5^, and road transportation^6^ has led to a significant enrichment in heavy metals (HMs) in soils. Excessive amounts of HMs in the soil environment can disrupt soil functions^7^ and cause risks to human health through ingestion, inhalation and dermal contact^8^. For example, Pb, Cd and Hg can cause damage to the human nervous system and kidneys, Cu can cause neurological problems and liver disease, and Ni can inhibit the development of immune organs by inducing excessive apoptosis and inhibiting cell proliferation^9^. The risks posed by HMs to human health and terrestrial ecosystems have attracted widespread attention, and HMs have been listed as priority pollutants for monitoring^10^. Therefore, a systematic study of HMs in the soil environment is of great importance for maintaining ecological security and human health.

Mountains are the starting point for materials circulation and energy flow in global ecosystems, and they are sensitive to global climate and environmental change^11^. Due to the vulnerability and sensitivity of the mountain ecological environment in arid areas, mountain systems are more likely to respond to external environmental changes, and soil contamination in mountain areas by HMs can reduce the service functions and values of mountain ecosystems and even pose threats to human health^12^. The rapid urbanization and industrialization that has occurred in recent decades have accelerated the accumulation of HMs in the environment^13^. Although mountainous areas are far from urban industrial centers that release HMs, the barrier effect and condensation effect of mountainous areas induce the deposition of HMs discharged from industries into mountainous soils through long-distance atmospheric transport^14^, making environmental safety issues increasingly prominent^15^. Scientific publications have shown that the development of transportation^16^ and tourism^17,18^ has also become a key factor in the enrichment of HMs in some mountainous areas. These results demonstrate that the sources of HMs in mountain soils are diverse; therefore, quantitative and qualitative analyses of the sources of soil HMs are particularly significant for precisely controlling pollution sources^19^. Presently, many quantitative methods are widely applied to identify sources of soil HMs, such as the PMF model^20^, principal component model analysis/multiple linear regression (PCA/MLR)^21^, UNMIX model^22^, and chemical mass balance method (CMB)^23^. Qualitative methods such as PCA, correlation analysis and cluster analysis cannot provide the contributions of pollution sources to HMs. Additionally, the stable isotopic compositions of HMs have an excellent tracing capacity, and stable isotopes of Pb, Zn, Cd, and Cu have been widely used for contamination source identification^23,24^. In the above approaches, PMF is a receptor model recommended by the U.S. Environmental Protection Agency (US EPA) for pollutant source apportionment, which takes into account the uncertainty of the data matrix and provides contributions of pollutant sources to HMs under nonnegative constraints^25^. The PMF model has been applied to source apportionment research of pollutants in the atmosphere, sediment and soils^26,27^ and has achieved positive results. Receptor models combined with isotopes are expected to be an efficient way to identify HM sources when multiple HMs coexist.

**Figure 1:**
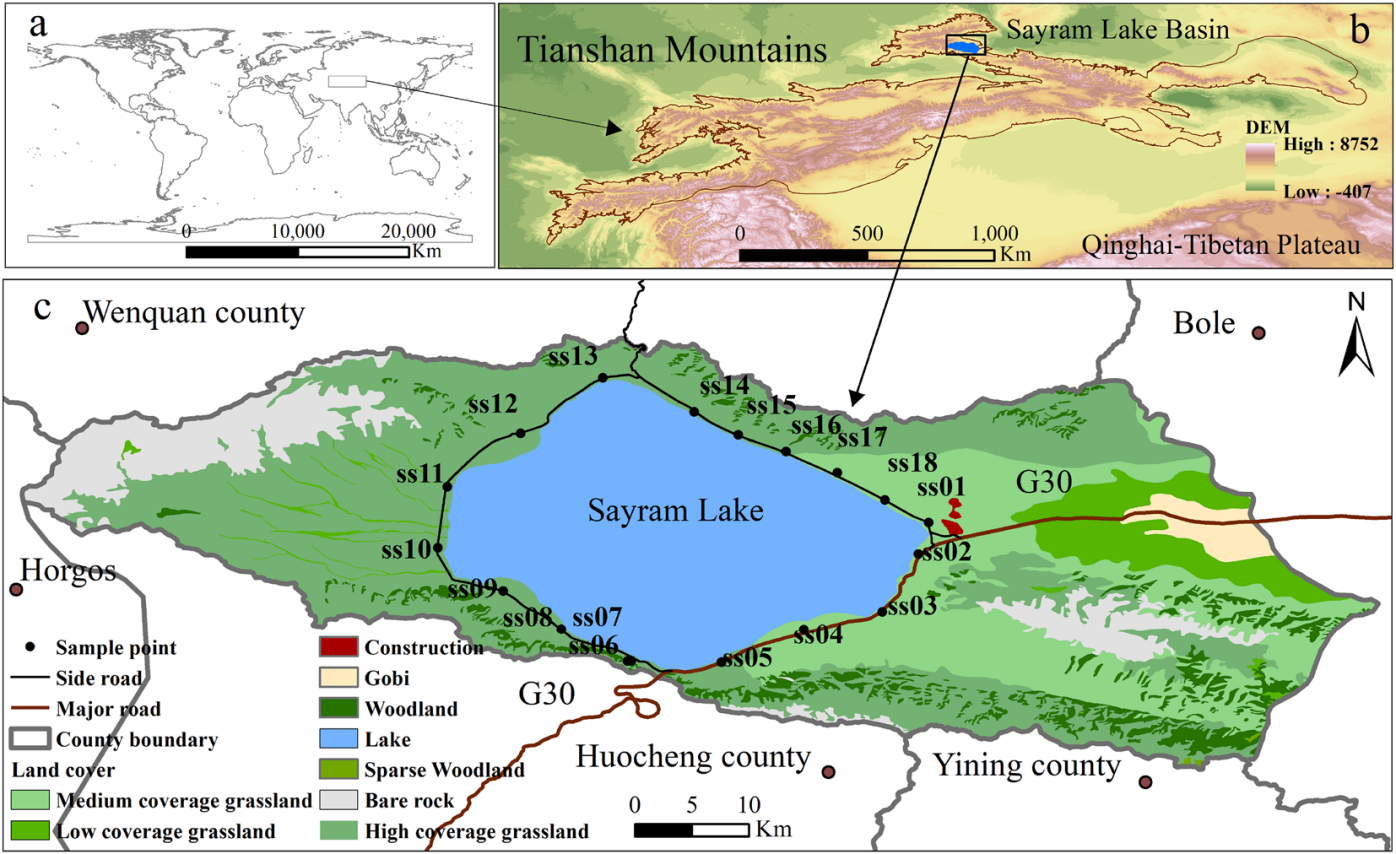
Location map and distribution of sampling points for the Sayram Lake Basin. The graphs (**a**, **b**) and (**c**) were generated by QGIS 3.26.3 (https://www.qgis.org) with the global vector data from the GADM database (https://gadm.org), the DEM data from the Natural Earth database (https://www.naturalearthdata. com), and the land use data from the Resource and Environmental Science and Data Center database (https://www.resdc.cn). The combination of graphs (**a**, **b**) and (**c**) was accomplished with linkscape 1.2.1 (https://inkscape.org).

The Sayram Lake Basin (Fig.1) is located in the middle of the West Tianshan Mountains in Xinjiang, which is a typical, closed watershed of the Tianshan Mountains. In recent decades, with economic development, tourism and transportation industries have rapidly emerged. As a 5A-level tourist attraction, the tourism area of Sayram Lake receives 2,530,000 tourists annually (http://www.xjboz.gov.cn/), which may cause high-intensity traffic problems and generate large amounts of tourist wastes, which lead to negative effects on the environment. However, these problems have not received sufficient attention. Existing studies on the Tianshan Mountains have focused on climate change and water resources^28,29^, land use and land cover^30^, snow and glaciers^31^, and paleolimnology^32^. The lack of research on the concentration, contamination and eco-risk characteristics of HMs and their influencing factors in the Tianshan Mountains has significantly affected the development of agriculture and pastoralism, tourism value evaluations and identifications of the ecological security of this region.

Thus, an investigation was carried out on the topsoil of the tourist area of Sayram Lake. The main objectives of this work were (1) to reveal the concentration characteristics of HMs; (2) to assess the degree of contamination and eco-risk of HMs; and (3) to identify the potential sources and contributions of soil HMs based on the PMF model and Pb isotope ratios. The results of the study are expected to provide scientific support for the prevention and control of HM pollution, for the sustainable development of the ecological environment and for the evaluation of ecological service values of mountain areas.

## Results

### Whole-rock geochemical compositions and grain-size characteristics

**Figure 2:**
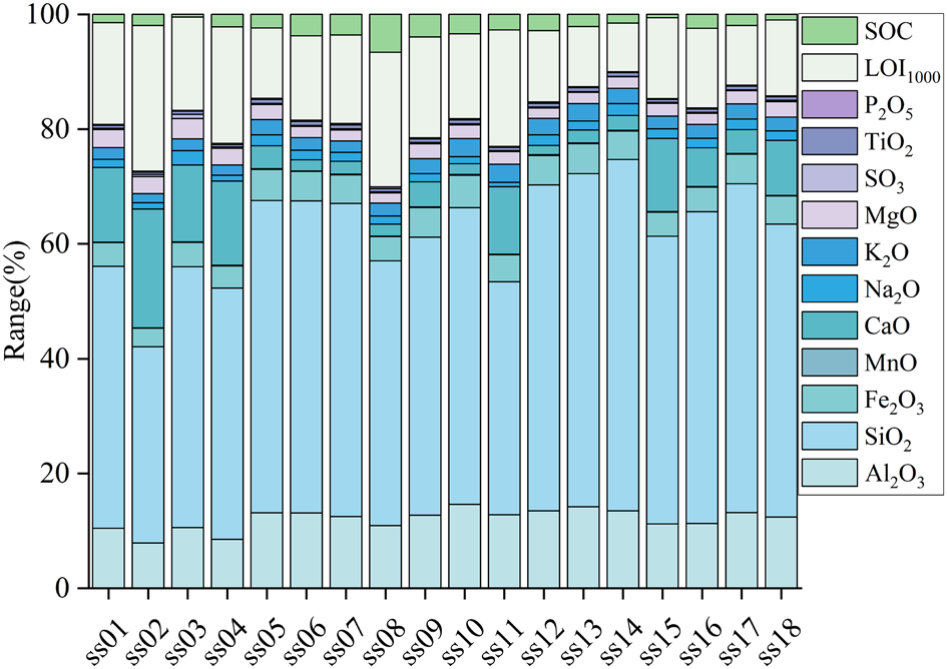
Whole-rock composition results of each sampling point in in the tourist area of Sayram Lake.

The whole-rock composition results are shown in Table S1, and the statistical characteristics are shown in Fig. 2. In general, the concentration range (%) and mean value (%) of the whole-rock composition in the topsoil of the tourist area of Sayram Lake decreased in the following order: SiO_2_ (34.8–62.09, 51.69) > LOI_1000_ (8.57–25.83, 16.02) > Al_2_O_3_ (7.99–15.18, 12.33) > CaO (1.71–21.10, 7.35) > SOC (2.61–10.50, 5.17) > Fe_2_O_3_ (1.6–3.3, 4.8) > K_2_O (1.6–3.33, 2.50) > MgO (1.85–3.57, 2.44) > Na_2_O (0.79–2.51, 1.61) > TiO_2_ (0.41–0.72, 0.61) > SO_3_ (0.10–0.73, 0.23) > P_2_O_5_ (0.14–0.35, 0.20) > MnO (0.06–0.15, 0.11).

**Figure 3:**
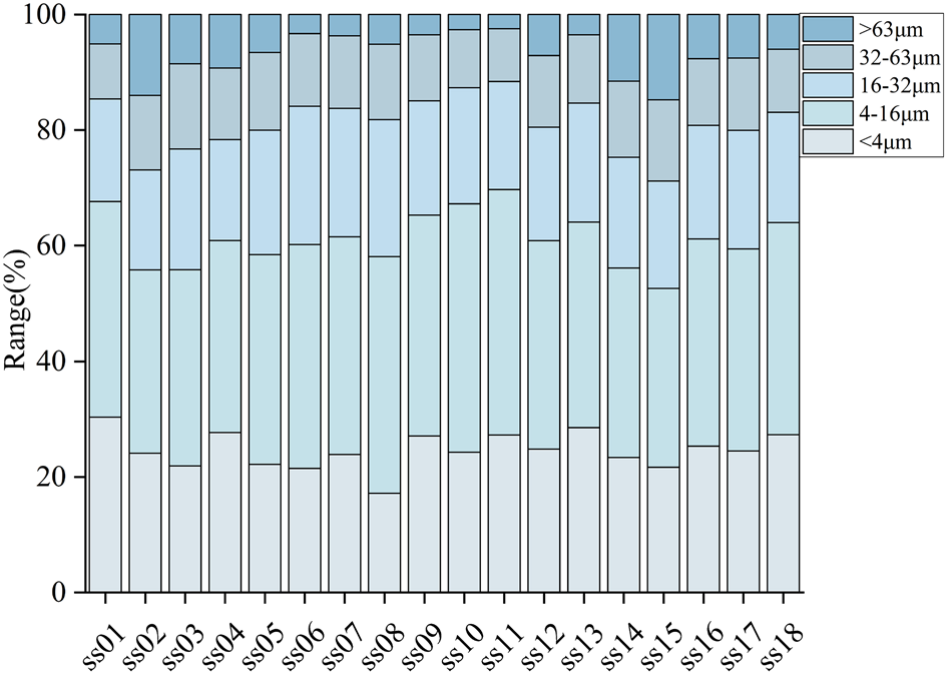
The grain-size composition of the topsoil in the tourist area of Sayram Lake.

The grain-size composition characteristics of the topsoil samples are shown in Fig. 3. According to the Udden-Wentworth classification^33^, the soil grain size was divided into five classes: clay (< 4 μm), fine-silty (4–16 μm), silty (16–32 μm), coarse-silty (32–64 μm) and sandy (> 64 μm). The percentage of grain-size composition of each level from large to small was fine-silty (36.49%) > clay (24.64%) > silty (19.98%) > coarse-silty (12.11%) > sandy (6.78%).

### Heavy-metal concentrations and Pb isotope ratios

**Figure 4:**
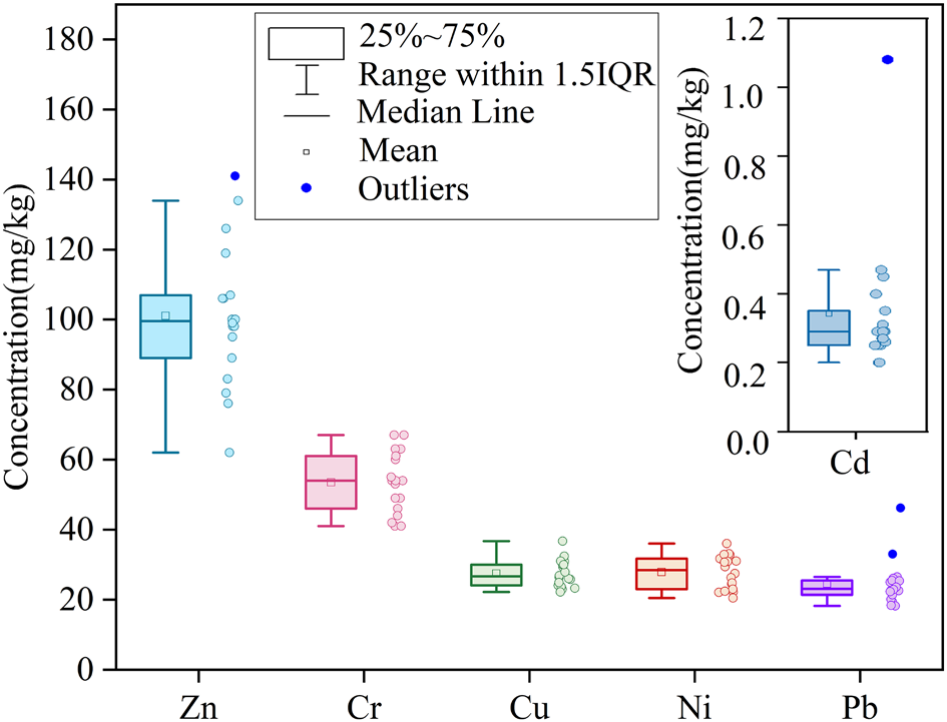
Concentrations of HMs in the topsoil of the study area.

The descriptive statistics for Cr, Cu, Ni, Pb, Zn and Cd in the topsoil of the tourist area of Sayram Lake are shown in Table S2, and the statistical characteristics are shown in Fig. 4. The highest concentration of HM was that of Zn, which ranged from 62–141 mg/kg, and the mean value was 101 mg/kg. The lowest concentration of HM was that of Cd, which ranged from 0.2–1.08 mg/kg, with an average value of 0.34 mg/kg. The average concentrations of Cr, Cu, Ni and Pb were 53.5 mg/kg, 27.48 mg/kg, 27.89 mg/kg and 24.59 mg/kg, respectively. Additionally, the average Pb and Zn concentrations exceeded the background values by 33% and 50%, respectively. The mean concentration of Cd was 2.8 times that of the background value (0.12 mg/kg). Furthermore, the CVs of Cd, Pb and Zn were 0.58, 0.26 and 0.2, respectively, while the CVs of the other elements were less than 0.2. These results demonstrated that Cd, Pb and Zn had strong variability in their mean values, and the variability in Cd was most significant.

The descriptive statistics of the Pb isotope ratios (^206^Pb/^207^Pb and ^208^Pb/^206^Pb) in the topsoil of the tourist area of Sayram Lake are shown in Table S2. The results showed that ^206^Pb/^207^Pb ranged from 1.09 to 1.21, with an average value of 1.15. ^208^Pb/^206^Pb ranged from 2.01 to 2.18, with an average value of 2.11.

### Contamination and eco-risk evaluation of HMs

The accumulation of HMs in soils may have a negative impact on the ecological environment. Therefore, researchers evaluate the effects of HMs on the environment based on a certain system of evaluation criteria and corresponding mathematical models. A single evaluation method may lead to errors in the results, so multiple evaluation criteria (*I*_*geo*_, PERI and PLI) were applied to evaluate the topsoil in the tourist area of Sayram Lake to strengthen the credibility of the assessment results.

The average *I*_*geo*_ values of HMs in the topsoil of the tourist area of Sayram Lake decreased in the following order: Cd (0.80) > Zn (−0.03) > Cu (−0.19) > Pb (−0.21) > Ni (−0.44) > Cr (−0.70). From the average value of *I*_*geo*_, the area was slightly contaminated with Cd (0.8), and the average value of *I*_*geo*_ of the other elements was less than 0, showing no contamination. From a more microscopic point of view, 22.2% of all sample points were moderately Cd-contaminated, these points were concentrated in the southeast of the study area (ss04, ss05, ss06, ss07), and the rest of the sample points were slightly contaminated. For Pb, there were two sample points with moderate contamination, and the *I*_*geo*_ of the remaining points was less than 0. For Zn, 39% of the samples were slightly contaminated. The *I*_*geo*_ values of Cr, Ni and Cu were less than 0, indicating that these HMs caused no contamination of the tourist area of Sayram Lake.

*E*^i^_r_ can indicate the risk of individual HMs. Referencing the grading criteria for *E*^i^_r_, Cr, Cu, Ni, Pb and Zn showed values of *E*^i^_r_ that did not exceed 40, indicating that they posed low risks. The mean *E*^i^_r_ of Cd was 85.69, which indicated a considerable risk. The PERI of Cd ranged from 67.39 to 295.80, with only one sample site (ss04) having a PERI (295.80) greater than 150, which was a moderate ecological risk area. The PLI ranged from 1.04 to 1.89, suggesting a moderate contamination level in the tourist area of Sayram Lake.

## Discussion

**Figure 5:**
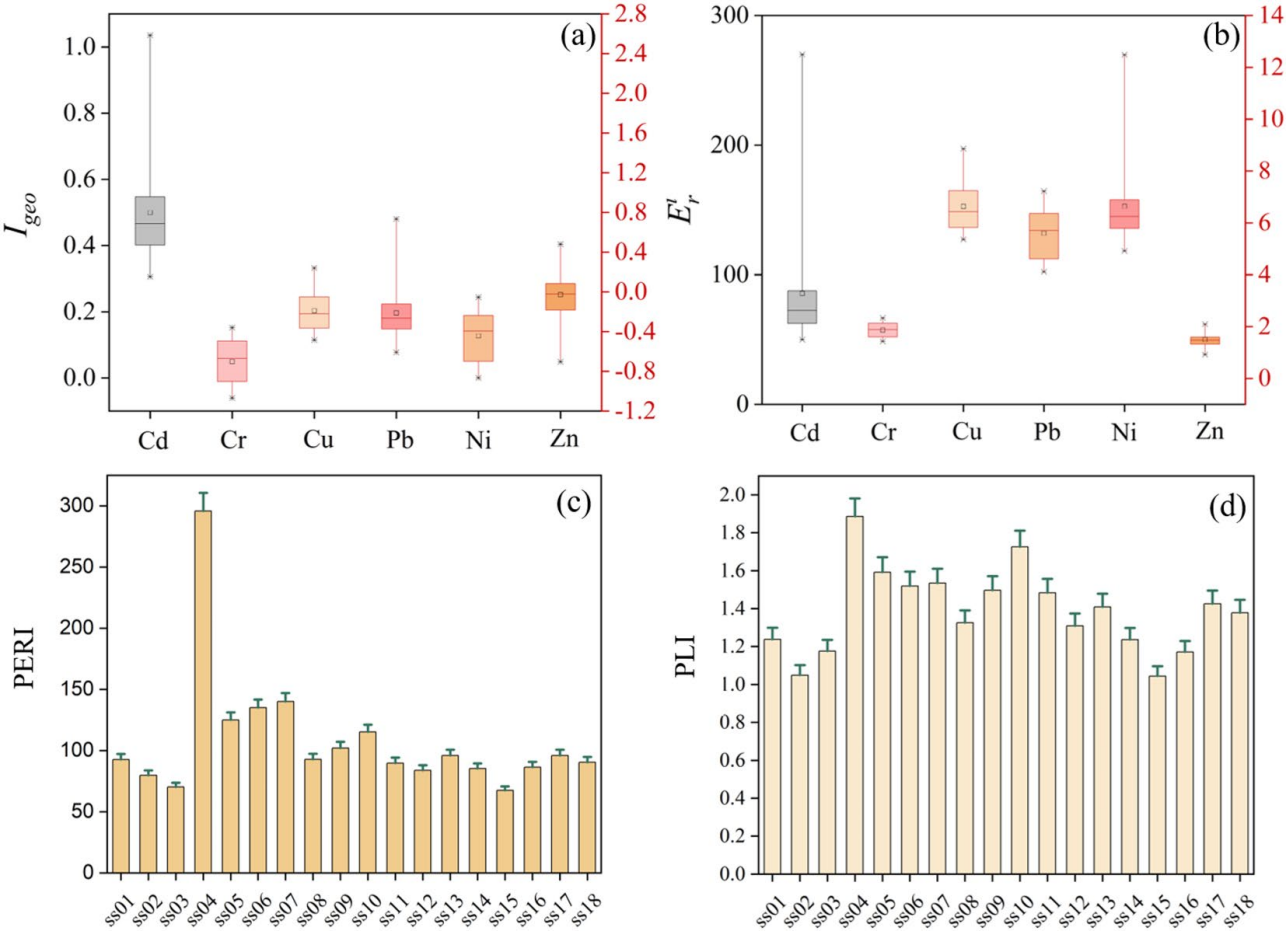
Contamination and ecological risk indices: (**a**) geoaccumulation index (*Igeo*) of HMs; (**b**) ecological risk of individual HMs; (**c**) potential ecological risk index (PERI) of HMs; (**d**) pollution load index (PLI) of HMs.

The plots of *I*_*geo*_, PERI, and PLI of HMs in the topsoil of the tourist area of Sayram Lake (Fig. 5) reveal the degree of HM pollution and eco-risk in this study area on the one hand and, on the other hand, indicate the direction for the relevant agencies to target soil environmental protection and HM pollution prevention and control measures. In this study, the *I*_*geo*_ results showed that Cd was the most highly enriched HM, and Pb, Zn, Cd, and Ni were slightly enriched in a few sample sites. The unnatural accumulation of these elements is usually closely associated with human activities in the area^34^. Tourism is the main economic activity in the district, and published studies have reported that tourism infrastructure construction (e.g., roads, buildings, etc.) and tourism wastes (e.g., plastic bags, batteries, hotel wastewater) release Cd into the soil^35^. Additionally, the accumulation of Pb, Zn, Cu and Ni in soils is usually associated with traffic emissions^36^. The PERI showed that the study area was at low risk overall, with only point ss04 exhibiting medium risk; however, this result was caused by the abnormally high Cd concentration value (Fig. 4) at point ss04 (Cd (concentration): 1.08 mg/kg, Cd (background): 0.34 mg/kg). This anomalous concentration value has a large influence on the PERI calculated based on the measured concentration, the background value and the toxicity coefficient. Therefore, references to this point can be appropriately removed when considering eco-risk. The PLI of each sampling point was greater than 1 and less than 2, which means that the area was in a moderately contaminated state. In general, the degree of soil HM contamination in this area was low; however, due to HM toxicity, bioaccumulation, and persistence^37^, the HM contamination of this area still requires sustained attention.

Correlation analysis is an efficient way to reveal correlations among HMs through Pearson correlation coefficients, and HMs with significant correlations may originate from the same source^38^. As shown in Table S5, the elemental pairs Cd-Cu (p < 0.01), Cd-Ni (p < 0.01), Cd-Zn (p < 0.05), Ni-Cu (p < 0.01), Pb‒Zn (p < 0.01), Cr-Ni (p < 0.01), Cr-Pb (p < 0.05), Cr-Zn (p < 0.05), Zn-Ni (p < 0.05), Zn-Cu (p < 0.01) were significantly and positively correlated. These results suggest that the elemental group Cd-Cu-Ni-Zn can be considered the same source. The significant correlation of Pb‒Zn implies that they have homologous characteristics, and published studies have reported that Pb and Zn are usually enriched by traffic emissions^20^. However, Cr, Ni, Pb and Zn may also share similar sources, and this result is probably caused by mixed sources of HMs, since natural sources, human activities in the study area, or atmospheric deposition may contribute to HM concentrations. Conversely, Cd showed no correlation with Cr and Pb, which indicated that they were controlled by different sources. Therefore, the PMF model was employed to further identify the sources of HMs and to quantify the contribution of each source to each HM.

The HM concentration data file and uncertainty data file were used as input data according to the EPA PMF5.0 user's guide. To ensure the accuracy and reasonableness of the model results, the factors were set to 2, 3 and 4, and the random starting seed number of the default model was run 100 times. It was determined that the explanatory rate of the model was best with 3 factors. In addition, the coefficient of determination *R*^2^ for all observed and predicted values of HMs was greater than 0.7, with a minimum value of 0.74 and a maximum value of 0.99. Bootstrapping (BS) and displacement of factor elements (DISP) were employed to evaluate the bias and uncertainty of the PMF results^39^. The results showed that over 90% of the base factors were reproduced in the BS model, and no factor swaps were observed in the DISP model within the lowest maximum permitted change in Q (*dQ*_*max*_). Therefore, it is reasonable and valid for the PMF model to explain the information contained in the original data using 3 factors.

**Figure 6:**
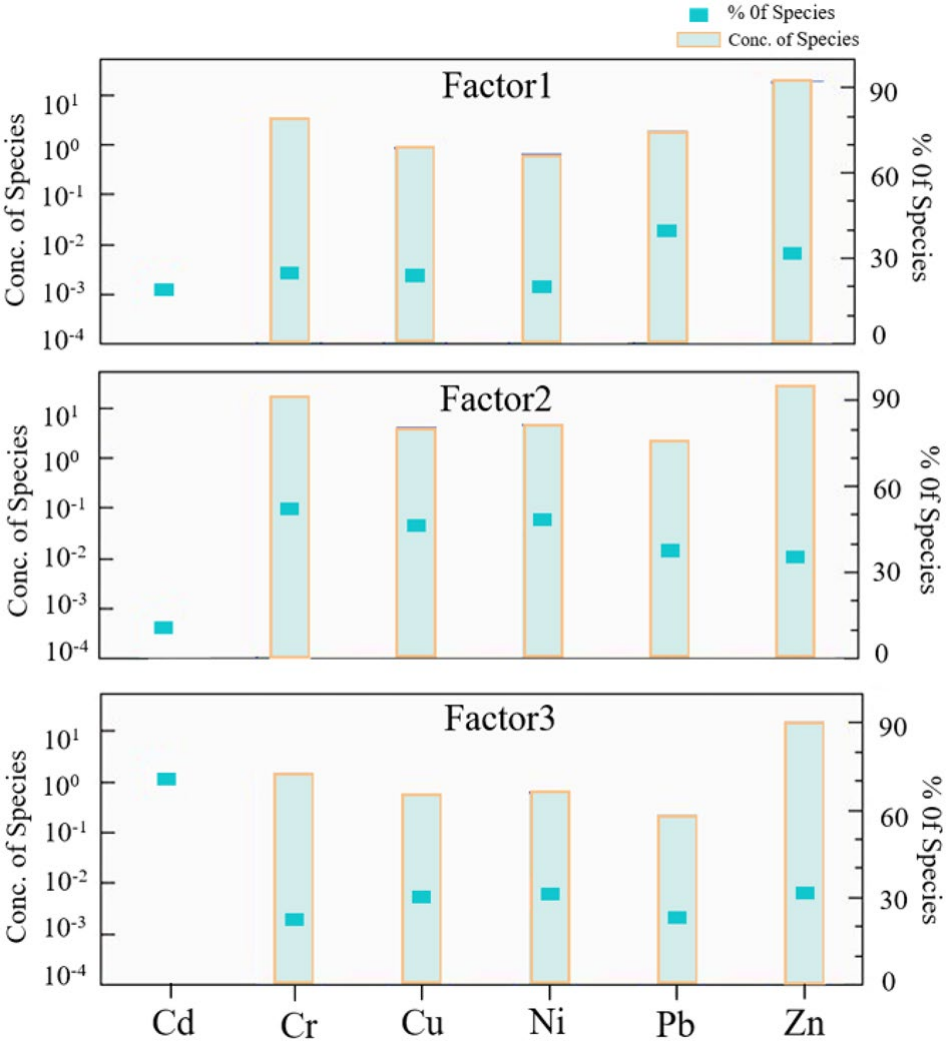
Sources and contributions of HMs in the soil in the PMF model.

The PMF model source apportionment results are shown in Fig. 6, where Factor 1 has high loading values for Pb and Zn (39.7% and 32.0%, respectively), implying that they were influenced by the same sources. The mean concentrations of Pb and Zn exceeded the background values for the tourist area of Sayram Lake, while the CVs were 0.26 and 0.2, respectively, which were medium variation levels, indicating that they might be influenced by human activities^40^. In addition, the *I*_*geo*_ indicated the presence of slight level of Pb and Zn contamination at some sampling points in the tourist area of Sayram Lake, which suggests the existence of external sources of Pb and Zn. Published studies have shown that Pb emissions from vehicle exhaust account for two-thirds of total global Pb emissions^41^; despite the global ban on the production and use of leaded gasoline since 2000, vehicle exhaust emissions are still considered to be the main cause of Pb accumulation in soils^42^. Additionally, the wear and tear of brakes, bearings and tires of automobiles also promote the release of Pb and Zn into the soil environment^43^. The soil samples collected in this study were located in the tourist area of Sayram Lake, where tourist visits reach values of 2,530,000 per year and may contribute significantly to local traffic. The frequent vehicle traffic on the G30 highway, emissions from vehicle exhaust and the wear and tear of parts may promote the deposition of Pb and Zn in the topsoil. Therefore, Factor 1 was inferred to be a traffic source.

Factor 2 was dominated by Cr, Cu, Ni, Pb, and Zn, with loadings of 52.2%, 45.8%, 48.9%, 37.1%, and 36.1%, respectively. The average concentration of Cr was lower than the background value, and the average concentrations of Cu and Ni slightly exceeded the local background values. Furthermore, they have small coefficients of variation, indicating low influence from external pollution sources. Notably, Pb and Zn showed high loadings in both Factor 1 and Factor 2, indicating that Pb and Zn were from mixed sources and were controlled by both Factor 1 and Factor 2. Previous studies have shown that Cr, Cu and Ni in soils commonly originate from the soil parent materials and are controlled by the geological background material and soil-forming processes^44^. Many studies also support this view^45,46^. Therefore, Factor 2 was inferred to be a natural source.

Factor 3 was mainly characterized by Cd, Ni, Cu and Zn, with loadings of 71.1%, 31.0%, 29.9% and 31.9%, respectively. In this study, the mean concentration of Cd was 2.8 times the local soil background value, with a CV of 0.58, which was a strong level of variation in relation to the mean, indicating that Cd may be influenced by anthropogenic sources. According to previous studies, the accumulation of Cd in soils is usually associated with agricultural activities (sewage irrigation, pesticide spraying and fertilizer use)^47^, plating of automotive lubricants and brake pads^48^, coal combustion^44^ and industrial ore smelting^49^. In addition, researchers have found that tourism activities can also increase levels of HMs in soils. In a study conducted by Wang et al.^50^ on the effect of tourism activities on soil quality in the scenic area of Mount. Tai, Shandong Province, it was found that tourism activities accelerated the accumulation of HMs, with the average content of Cd exceeding the background value by 36.2%. Enrichment of HMs in soils under tourism loading conditions was also reported in another study, where local soils were highly contaminated with Cd^4^. An investigation of the study area showed that there were no mineral smelting activities or agricultural activities in the basin. The enrichment of Cd may originate from HM-containing wastes from tourism activities (batteries, plastics, wastewater, etc.) and atmospheric deposition (e.g., deposition from the wear and tear from motor vehicles and combustion of coal for heating). Therefore, Factor 3 can be considered a mixed effect of the above sources.

Figure S1 shows the contribution of three factors (F1: traffic source, F2: natural source, F3: tourist waste and atmospheric deposition) to HMs. The contributions of the three factors to Pb and Zn in the soil in a descending order were F1 ≈ F2 > F3. Cr, Cu and Ni were mainly influenced by F2. Cd was controlled by F3 (71.1%). Overall, it seems that the HMs of the topsoil in the tourist area of Sayram Lake were influenced by multiple sources, with the greatest contribution from F2 (38.5%), followed by F3 (34.3%) and F1 (27.2%). Notably, F3 has the greatest capacity to release Cd into the soil environment, and F3 control should be considered a priority.

**Figure 7:**
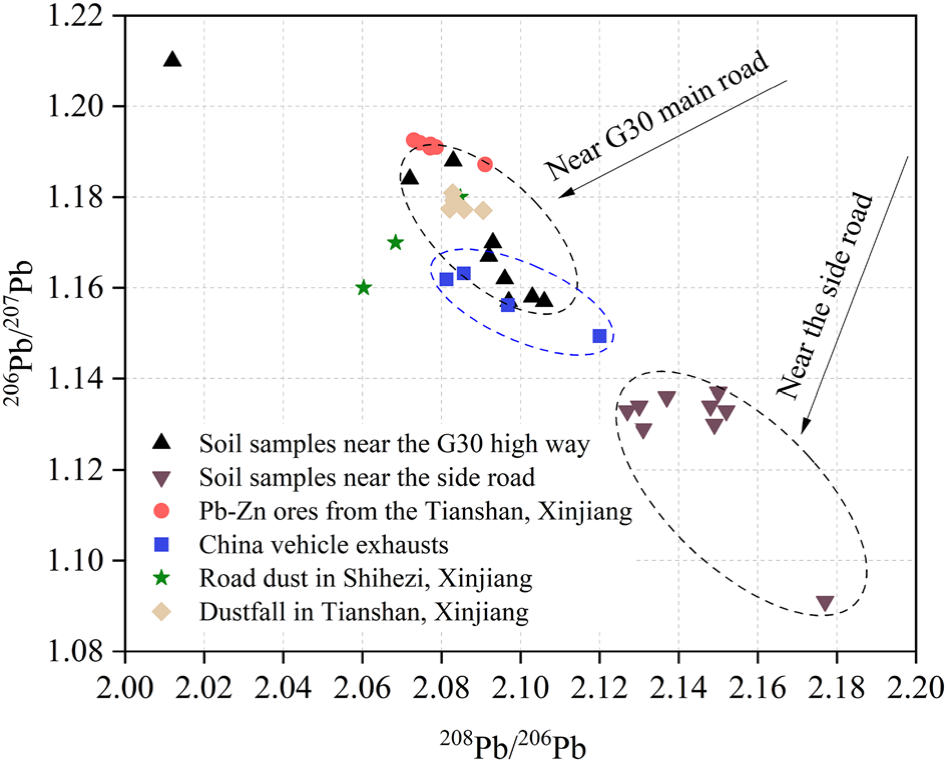
Comparison of Pb isotopic ratios in the topsoil with other relevant studies reported.

To validate the results of the PMF model, the sources of Pb in the soil were further analyzed using the Pb isotope method. The Pb isotope ratio results confirmed that the Pb present in the topsoil of the tourist area of Sayram Lake was influenced by anthropogenic sources. As shown in Fig. 7, the Pb isotope ratios in soil samples from the tourist area of Sayram Lake were plotted with those in other related environments (^206^Pb/^207^Pb vs. ^208^Pb/^206^Pb). The Pb isotope ratio data of relevant environments mainly include Pb‒Zn ores from the Tianshan Mountains in Xinjiang^51^, vehicle exhaust emissions in China^52,53^, urban road dust in Xinjiang^54^ and dustfall in the Tianshan Mountains^55^. Figure 7 shows that there were significant differences in the Pb isotope ratios of different regions. The sampling points near G30 had higher ^206^Pb/^207^Pb ratios and lower ^208^Pb/^206^Pb ratios. The sampling points near side roads had lower ^206^Pb/^207^Pb ratios and higher ^208^Pb/^206^Pb ratios. The Pb isotope ratios at the sampling sites located near the G30 main road were similar to those of vehicle exhaust emissions, urban road dust and dustfall, and the results indicated that Pb enrichment was strongly related to traffic emissions. Increased human activity has accelerated the accumulation of HMs in the environment, and isotopic methods can identify the sources of HMs based on the similarities they exhibit^14^. By integrating the Pb isotope data in each environment, it was determined that the Pb in the soil of the tourist area of Sayram Lake was partially derived from traffic emissions. This result validated the plausibility of factor 1 in the PMF model being a traffic source.

A comparison with other tourist areas (Table 1) yielded two distinctive findings: Pb, Zn, Cd and Cu are common pollutants that can easily accumulate in soils in tourism areas; the enrichment of HMs is strongly related to the type of local land use and human activities. Differences in human activities and the geographic contexts of each area lead to differences in the influencing factors and enrichment levels of HMs^56^. To safeguard the soil environmental, HMs that are easily enriched in tourist areas should be highlighted for monitoring, and anthropogenic sources around tourist areas should be reasonably controlled.

**Table 1:** Comparison of the main pollutants and sources in the topsoil of the Sayram Lake tourist area and other tourist research areas.

Study area	Properties	Major pollutants	Driving factors	References
Sayram Lake basin (China)	Tourist area	Cd Pb Zn	Transportation; Tourist waste; Atmospheric deposition;	This study
Dongting lake region (China)	Tourist area	Cd Pb Zn	Transportation; Household waste; Agriculture; Industry	^72^
Issyk-Kul lake basin (Kyrgyzstan)	Tourist area	Cd Pb Zn	Atmospheric Deposition; Agriculture	^73^
Bishkek (Kyrgyzstan)	Tourist area	Pb Zn Cu	Transportation	^74^
Zakopane (Poland)	Tourist town	Cd Zn Pb	Coal combustion; Transportation	^75^
Gui lin (China)	Tourist town	Pb Zn	Fuel combustion; Transportation	^76^
Vesuvius (Italy)	National Park	Pb Cu	Transportation	^77^
Huangshan (China)	National Park	Cd	Transportation	^78^

**Figure 8:**
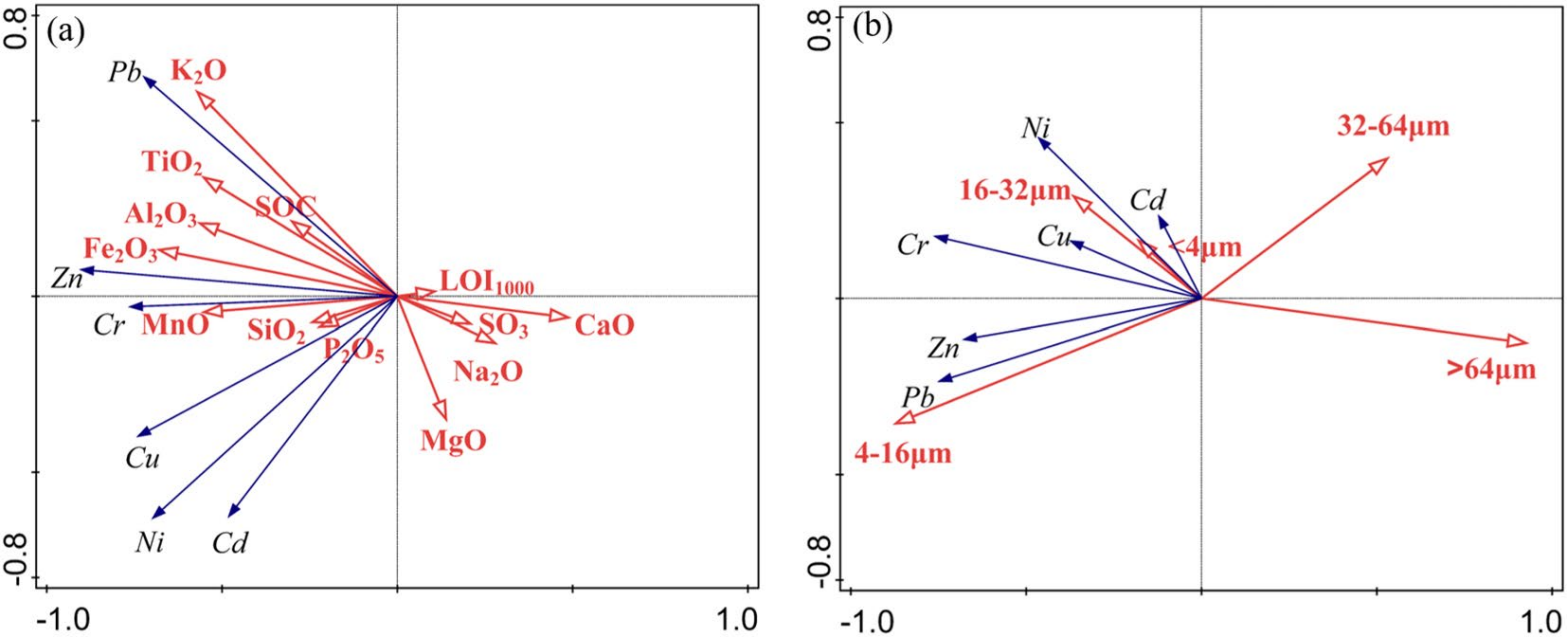
Redundancy analysis of HMs and whole-rock composition, soil organic carbon, and soil grain-size composition.

The material composition and structure of the soil influence the concentration of HMs in soils. To investigate the effect of soil composition and structure on HMs under natural conditions, data including whole-rock composition, grain size, soil organic carbon and loss-on-ignition were used for redundancy analysis with HMs. Figure 8(a) reveals the relationship between SiO_2_, LOI_1000_, Al_2_O_3_, CaO, Na_2_O, TiO_2_, SO_3_, P_2_O_5_, MnO, K_2_O, Fe_2_O_3_, MgO, SOC and Cd, Cr, Cu, Ni, Pb and Zn. The results showed that the content of HMs was related to SiO_2_, Al_2_O_3_, TiO_2_, P_2_O_5_, MnO, K_2_O, Fe_2_O_3_ and SOC. Among them, Pb was most closely related to Al_2_O_3_ and SOC, and previous studies have shown that soil organic matter has a strong adsorption capacity for HMs^57^. Zn, Cr, Cu and Ni were closely related to Fe–Mn minerals and silicates, which was similar to the results of Ma et al.^58^. Additionally, this also indicated that HMs were weakly affected by carbonate and sulfate. The relationship between HMs and grain size (Fig. 8b) showed that HMs tended to be stored in soil particles with grain sizes < 32 µm. Among them, Cd, Ni, Cu and Cr were associated with clay (< 4 µm) and silt (16–32 µm), while Pb and Zn were associated with a fine-silty (4–16 µm) grade.

The findings revealed that the enrichment of Cd, Pb and Zn in the topsoil of the tourist area of Sayram Lake was influenced by anthropogenic sources. The results of the contamination and eco-risk evaluation suggest that the potential risks posed by the high *I*_*geo*_ and *E*^i^_r_ values of Cd should be treated seriously by relevant authorities. Intensive visitors and traffic will promote the release of HMs that accumulate in the topsoil to contaminate the soil environment and weaken its functions. Tourism quotas have been suggested to reduce soil contamination and degradation^17^. However, for traffic emissions of HMs beyond individual or regional control (e.g., lead in gasoline, car brakes, brake pads and tires), authorities may change their approach and undertake soil remediation. For example, the hyperaccumulation function of plants can be used to reduce HMs in soils or appropriate amounts of soil conditioners can be added^59^ to reduce the polluting capacity of HMs by agglomerating them through adsorption and complexation effects.

In this study, the concentration characteristics, potential sources, contamination levels and eco-risk assessment of HMs were analyzed by collecting topsoil samples from the tourist area of Sayram Lake, but some limitations exist. First, a small number of topsoil samples were collected in this study; therefore, they might not adequately reflect the environmental status of the study area and reveal the spatial distribution characteristics of HMs. In addition, the objects of this study only involved Cd, Cr, Cu, Ni, Pb and Zn in the soil, and the absence of studies on other HMs (e.g., Hg, Co, W, etc.) and metalloids (As) has limitations for gaining an overall understanding of the soil environment in this study area. Additionally, HM source apportionment analysis should be combined with multiple source analysis methods to avoid contingency and subjectivity of the results. For example, quantitative analysis methods such as PCA-MLR and CMB can be combined to investigate potential sources and contributions of HMs, and isotope tracing methods for Cd, Cu and Zn can be used to reveal the sources of HMs more accurately. It is worth noting that there are some differences in the pollution evaluation results of the PERI and PLI methods that were caused by the different evaluation standards and thresholds adopted by the different methods. Therefore, the regional applicability of pollution evaluation methods is challenging, and subsequent studies on the toxicological effects of HMs in the region could be carried out to determine the pollution evaluation criteria and thresholds suitable for this region.

## Conclusions

This study examined Cd, Cr, Cu, Ni, Pb and Zn in the topsoils of the Sayram Lake tourist area in Xinjiang, discussed their concentration characteristics, contamination levels, eco-risk characteristics and potential sources, and reached the following conclusions:

1. Cd, Pb and Zn were generally enriched in the topsoil, and their mean concentrations were 2.83, 1.33, and 1.5 times higher than the local background values, respectively. In contrast, the average concentrations of Cr, Cu and Ni were close to the corresponding background values.
2. The calculated *I*_*geo*_ and *E*^i^_r_ indicated that the topsoil was more severely contaminated with Cd. The PERI suggested that the eco-risk in this study area was low, and the PLI in the tourist area of Sayram Lake ranged from 1.04 to 1.89, with a mean value of 1.39, indicating a moderate contamination level.
3. Three sources of soil HMs were identified and apportioned by combining the PMF model and Pb isotope ratios, namely, traffic sources (27.2%), natural sources (38.5%), and mixed sources (tourism waste and atmospheric deposition) (34.3%). The source apportionment results showed that Cr, Cu, and Ni were mainly derived from natural sources, Pb and Zn were attributed to traffic sources and natural sources, and Cd was associated with tourist wastes and atmospheric deposition.
4. The utilization of whole-rock composition and grain size data of the soils was an efficient way to study the influencing factors for HMs. The content of HMs in the topsoil of the tourist area of Sayram Lake was influenced by SiO_2_, Al_2_O_3_, TiO_2_, P_2_O_5_, MnO, K_2_O, Fe_2_O_3_ and SOC. Additionally, the content of HMs was also influenced by soil grain size, and HMs tended to be stored in soil particles with grain sizes < 32 µm.

## Materials and methods

### Geographical setting

The Sayram Lake Basin (Fig. 1) is located on the northern slope of the Tianshan Mountains and in the hinterland of the Eurasian continent. The basin belongs to Bole city, adjacent to Wenquan County, Horgos city, Huocheng County and Yining County, with a drainage area of 1408 km^2^ ^60^. The area has a temperate alpine climate type, with an annual average temperature and an annual average precipitation of 0.5 °C and 350 mm, respectively^61^.

The vegetation type of the Sayram Lake watershed is relatively homogeneous, with the lake area making up 24.8% of the watershed area, and the rest of the land is mainly covered by high-, medium- and low-coverage grassland (59.6%), woodland (5.2%), bare rock (8.7%), and Gobi (1.5%) compositions (Fig. 1). The tourist area of Sayram Lake is popular among tourists, with an average daily admission of nearly 7,000 visitors. The G30 highway runs along the southeast side of Sayram Lake through the basin to bring superior transportation conditions, while the construction of a 79 km-long tourist road around the lake promotes tourism.

### Sample collection

To study the accumulation of HMs caused by human activities in Sayram Lake Basin, sampling sites were predefined in this study based on the traffic deployment in the Sayram Lake watershed and the activity areas of tourists in this tourist area. In total, 18 topsoil samples were collected from the study area, and topsoil samples were collected from 0–20 cm in October 2020, and GPS was used for geographic positioning. To prevent the topsoil samples from becoming cross-contaminated, the collected topsoil samples were immediately placed in polyethylene bags and sealed for storage. The stainless-steel drill bit was cleaned between samples.

### Sample preparation and laboratory analysis

Soil samples collected in the field were naturally dried at room temperature in the laboratory. Then, impurities were removed from the soil samples, and the soil was crushed using an agate mortar and passed through a 200-mesh nylon sieve. The processed soil samples were sealed in polyethylene bags for further chemical analysis. The determination and analysis of HMs and whole-rock, organic carbon and Pb isotopes in the soil samples were performed at ALS Minerals-ALS Chemex (Guangzhou, China).

The soil samples (0.125 g) were digested in three stages using HCl-HNO_3_-HF-HClO_4_. First, HNO_3_ and HClO_4_ were used for preoxidation. Then, HF was added, and the mixture was heated in an electric furnace. Finally, the remaining solution was diluted with HCl. The concentrations of Cr, Cu, Ni, Pb and Zn were determined by inductively coupled plasma atomic emission spectroscopy (ICP‒AES) (Agilent, 5110, USA). The concentrations of Cd were determined by inductively coupled plasma‒mass spectrometry (ICP‒MS) (Agilent, 7900, USA).

An X-ray fluorescence spectrometer (PANalytical, PW2424, Netherlands) was used to analyze the whole-rock composition of the soil, and the ME-XRF26 method provided by ALS Minerals-ALS Chemex (Guangzhou, China) was applied to determine the Al_2_O_3_, CaO, Fe_2_O_3_, K_2_O, MgO, MnO, Na_2_O, P_2_O_5_, SiO_2_, SO_3_ and TiO_2_ content percentages. The loss-on-ignition of soil samples was determined based on the OA-GRA05x method provided by ALS Minerals-ALS Chemex (Guangzhou, China) at 1000 °C (LOI_1000_). The detection limit for loss-on-ignition was 0.01%, and the relative deviation and relative error of the method were both less than 5%. A carbon–sulfur analyzer (LECO, CS844, USA) was used for soil organic carbon analysis. The soil samples that were digested by HCl were separated and filtered for organic carbon with a porous crucible. The crucible was cleaned with deionized water, dried and placed in an infrared induction furnace to quantitatively detect the percentage content of soil organic carbon. The detection limit for soil organic carbon was 0.02%, and the relative deviation and relative error of the method were less than 5% and 3.5%, respectively.

The Pb isotope ratios (^208^Pb/^206^Pb and ^207^Pb/^206^Pb) of the digested soil were determined by ICP‒MS (Agilent 7700x), and the instrument was calibrated using a reference material (SRM981-NIST, USA) and standard material (GBW04426, China) for quality control. The relative standard deviations (RSD) of ^208^Pb/^206^Pb and ^207^Pb/^206^Pb were less than 0.02 for multiple measurements of the reference material GBW04426.

Soil grain size was measured by a Bettersizer laser grain-size analyzer (BT-9300SE) manufactured in China, and the measurement accuracy error and repeatability error were both less than 1%. According to the Udden-Wentworth^33^ grain size classification standard, the soil grain size in this study included clay (< 4 μm), fine-silty (4–16 μm), silt (16–32 μm), coarse-silty (32–64 μm) and sandy (> 64 μm) grades.

### Quality assurance and quality control

To ensure the accuracy of the experimental data, monitoring materials (blank samples, duplicate samples and standard materials (MRGeo08, GBW07179) were inserted in each testing batch for HM testing of soil samples. The detection limits for HMs (Cr, Cu, Ni, Zn, Pb and Cd) were 1 mg/kg, 0.2 mg/kg, 0.2 mg/kg, 2 mg/kg, 0.5 mg/kg and 0.02 mg/kg, respectively, and the analytical errors were less than 5%. The limits of detection for whole-rock compositions (Al_2_O_3_, CaO, Fe_2_O_3_, K_2_O, MgO, MnO, Na_2_O, P_2_O_5_, SiO_2_, SO_3_ and TiO_2_) were 0.01%, and the analytical errors were less than 5%.

### Statistical analysis methods

In this study, R 4.0.4 software was used for descriptive statistics, descriptive data such as the mean, maximum and minimum values, and coefficient of variation (CV) were calculated, and Pearson correlation analysis was used for preliminary source analysis. Origin 2021b software was used to map the whole-rock composition and particle size characteristics, elemental concentration characteristics, ecological risk assessment results and comparative Pb isotopes of the soil samples. Canoco5^62^ was applied to complete redundancy analysis between HMs and whole-rock elements and grain size.

### Ecological risk assessment methods

(1) Geoaccumulation index (*I*_*geo*_)**.** To assess the degree of HM contamination in the topsoil of the tourist area of Sayram Lake, *I*_*geo*_ was used as an indicator for evaluation, and it was defined as follows^63^:

1 $${I}_{geo}={{\log}}_{2}\left(\frac{{C}_{i}}{1.5*{B}_{i}}\right)$$

where *C*_*i*_ represents the measured concentration value of element *i*, and *Bi* represents the local background value of element *i*. The background values of HMs in this study were referenced from the 1994 statistics of the China National Environmental Monitoring Centre^64^ in the Sayram Lake Basin (Cd: 0.12, Cr: 57.3, Cu: 20.7, Ni: 24.9, Pb: 18.5, Zn: 67.3, Unit: mg/kg). *I*_*geo*_ is divided into 7 categories^65^, indicating different levels of contamination, and the classification information is presented in Table S3.

(2) Pollution load index (PLI). The PLI method was originally developed by Tomlinson et al. and is now widely used for soil HM contamination assessment^66^. The calculation is as follows:

2 $$C{F}_{i}=\frac{{C}_{i}}{{B}_{i}}$$

3 $$PLI={\left(C{F}_{1}*C{F}_{2}*C{F}_{3}*\cdots *C{F}_{n}\right)}^\frac{1}{n}$$

where *CF*_*i*_ represents the pollution index value of a single factor, *Ci* denotes the measured concentration of pollutant *i* and *Bi* refers to the background value of element *i*. A PLI less than 1 indicates no contamination, and a PLI greater than 1 and less than 2 indicates moderate contamination.

(3) Potential ecological risk index (PERI). The PERI method was originally developed by Hakanson^67^, it integrates the ecological and toxicological effects of HMs, and it is widely used for the assessment of the eco-risk and contamination levels of the soil environment by HMs^68^.

4 $${C}_{r}^{i}=\frac{{C}_{i}}{{B}_{i}}$$

5 $${E}_{r}^{i}={T}_{i}*{C}_{r}^{i}$$

6 $$PERI=\sum_{i=1}^{n}{E}_{r}^{i}$$

where *C*_*i*_ denotes the measured concentration of pollutant *i*, *B*_*i*_ denotes the background concentration of element *i*, *Ci r* denotes the enrichment coefficient of the element, *E*^i^_r_ denotes the eco-risk of the individual pollutant, and *T*_*i*_ represents the toxicity coefficient, which was derived from a previously published study for Cd, Cr, Cu, Ni, Pb, and Zn (the values were 30, 2, 5, 5, 5, and 1, respectively)^69^. PERI is classified into 4 risk levels, ranging from low risk to significantly high risk (Table S4).

### Positive matrix factorization (PMF)

The PMF model is a receptor model recommended by the US EPA for source analysis of environmental pollutants^70,71^, which was used in this study for source analysis of HMs in the tourist area of Sayram Lake. The basic equation of the PMF receptor model is as follows:

7 $${X}_{ij}=\sum_{k=1}^{p}\left({g}_{ik}{f}_{kj}+{e}_{ij}\right)$$

where *X*_*ij*_ denotes the measured concentration of HM *j* in sample *i*, *g*_*ik*_ denotes the contribution of the *k*th source to the *i*th sample, *f*_*ik*_ represents the eigenvalue of contamination source *k* to the *j*_*th*_ HM concentration, and *e*_*ij*_ is the residual matrix. The optimal matrix of *g* and *f* can be obtained by continuously minimizing the objective function *Q*. The calculation of Q is as follows:

8 $$Q={\sum_{i=1}^{n}\sum_{j=1}^{m}\left(\frac{{e}_{ij}}{{u}_{ij}}\right)}^{2}$$

where *u*_*ij*_ is the uncertainty, and its calculation depends on whether the concentration of HMs is lower than the method detection limit (MDL). When the concentration of HMs exceeds the MDL, *u*_*ij*_ can be calculated according to the following equation:

9 $${u}_{ij}=\sqrt{{\left(error\, fraction*c\right)}^{2}+MD{L}^{2}}$$

Otherwise, *u*_*ij*_ is calculated by the following equation:

10 $${u}_{ij}=5/6*MDL$$

where *c* is the concentration of the HMs, MDL denotes the method detection limit, and the error fraction is the percentage of measurement uncertainty.

## Supplementary Information

Supplementary Information.

## Data Availability

The datasets generated and/or analyzed during the current study are not publicly available because the data are a part of an ongoing study, but they are available from the corresponding author on reasonable request.
