# Supplementary material for: Source apportionment of soil heavy metals with PMF model and Pb isotopes in an intermountain basin of Tianshan Mountains, China
Source: Sci Rep. 2022 Nov 12;12:19429. doi: 10.1038/s41598-022-24064-1 (PMC9653478; doi:10.1038/s41598-022-24064-1)
Supplement: Supplementary file 1 — Supplementary Information. [file 41598_2022_24064_MOESM1_ESM.docx]

**Source apportionment of soil heavy metals with PMF model and Pb isotopes in an intermountain basin of Tianshan Mountains, China**

**Tao Zeng ^1,2,3^, Long Ma ^1,2,3, *^, Yizhen Li ^1,2,3^, Jilili Abuduwaili ^1,2,3^, Wen Liu ^1,2,3^, Sen Feng ^1,2,3^**

*^1^ State Key Laboratory of Desert and Oasis Ecology, Xinjiang Institute of Ecology and Geography, Chinese Academy of Sciences, Urumqi 830011, China*

*^2^ Research Center for Ecology and Environment of Central Asia, Chinese Academy of Sciences, Urumqi 830011, China*

*^3^ University of Chinese Academy of Sciences, Beijing 100049, China*

* Correspondence: [malong@ms.xjb.ac.cn](mailto:malong@ms.xjb.ac.cn)

Table S1. Descriptive statistics of whole-rock geochemical composition of topsoil in the tourist area of Sayram Lake (Units: %).

|  | Al_2_O_3_ | SiO_2_ | Fe_2_O_3_ | MnO | CaO | Na_2_O | K_2_O | MgO | SO_3_ | TiO_2_ | P_2_O_5_ | LOI_1000_ |
| --- | --- | --- | --- | --- | --- | --- | --- | --- | --- | --- | --- | --- |
| Min | 7.99 | 34.80 | 3.28 | 0.06 | 1.71 | 0.79 | 1.60 | 1.85 | 0.10 | 0.41 | 0.14 | 8.57 |
| Max | 15.18 | 62.09 | 5.86 | 0.15 | 21.10 | 2.51 | 3.33 | 3.57 | 0.73 | 0.72 | 0.35 | 25.83 |
| Median | 13.03 | 52.61 | 4.99 | 0.11 | 4.40 | 1.64 | 2.44 | 2.29 | 0.20 | 0.63 | 0.18 | 15.33 |
| Mean | 12.33 | 51.69 | 4.81 | 0.11 | 7.35 | 1.61 | 2.50 | 2.44 | 0.23 | 0.61 | 0.20 | 16.02 |
| SE of mean | 0.46 | 1.67 | 0.16 | 0.01 | 1.39 | 0.10 | 0.11 | 0.12 | 0.03 | 0.02 | 0.01 | 1.11 |
| Standard Deviation | 1.93 | 7.07 | 0.67 | 0.03 | 5.91 | 0.40 | 0.47 | 0.50 | 0.14 | 0.09 | 0.05 | 4.72 |
| Coefficient of Variation | 0.16 | 0.14 | 0.14 | 0.25 | 0.80 | 0.25 | 0.19 | 0.21 | 0.61 | 0.15 | 0.25 | 0.29 |

Table S2. Descriptive statistics of heavy metal concentrations and Pb isotopes in topsoil of the tourist area of Sayram Lake.

|  | Cd | Cr | Cu | Ni | Pb | Zn | ^206^Pb/^207^Pb | ^208^Pb/^206^Pb | |
| --- | --- | --- | --- | --- | --- | --- | --- | --- | --- |
| Units | mg kg^-1^ | mg kg^-1^ | mg kg^-1^ | mg kg^-1^ | mg kg^-1^ | mg  kg^-1^ | - | | - |
| Minimum | 0.20 | 41.00 | 22.20 | 20.50 | 18.20 | 62.00 | 1.09 | | 2.01 |
| Maximum | 1.08 | 67.00 | 36.70 | 36.00 | 46.20 | 141.00 | 1.21 | | 2.18 |
| Median | 0.29 | 54.00 | 26.65 | 28.45 | 23.10 | 99.50 | 1.15 | | 2.12 |
| Mean | 0.34 | 53.50 | 27.47 | 27.88 | 24.58 | 101.00 | 1.15 | | 2.11 |
| SE of mean | 0.05 | 2.04 | 0.91 | 1.12 | 1.51 | 4.74 | 0.01 | | 0.01 |
| Standard Deviation | 0.20 | 8.67 | 3.86 | 4.75 | 6.42 | 20.12 | 0.03 | | 0.04 |
| Coefficient of Variation | 0.58 | 0.16 | 0.14 | 0.17 | 0.26 | 0.20 | 0.02 | | 0.02 |
| Background | 0.12 | 57.30 | 20.70 | 24.90 | 18.50 | 67.30 | - | | - |

Table S3. Grading standards for Geoaccumulation index (*I_geo_*)

| *I_geo_* | Description |
| --- | --- |
| *I_geo_*≤0 | uncontaminated |
| 0<*I_geo_*≤1 | slightly contaminated |
| 1<*I_geo_*≤2 | moderately contaminated |
| 2<*I_geo_*≤3 | moderately to heavily contaminated |
| 3<*I_geo_*≤4 | heavily contaminated |
| 4<*I_geo_*≤5 | heavily to extremely contaminated |
| 5<*I_geo_* | extremely contaminated |

Table S4. Grading standards for ecological risk assessment index

| *Ei r* | Description | PERI | Description |
| --- | --- | --- | --- |
| *Ei r* < 40 | Low ecological risk | PERI<150 | Low risk |
| 40 < *Ei r* ≤ 80 | Moderate ecological risk | 150<PERI<300 | Moderate ecological risk |
| 80 < *Ei r* ≤ 160 | Considerable ecological risk | 300<PERI<600 | High ecological risk |
| 160 < *Ei r* ≤ 320 | High ecological risk | PERI≥600 | Significantly high ecological risk |
| *Ei r* > 320 | serious ecological risk |  |  |

Table S5. Correlation coefficient matrix of soil heavy metal concentrations

|  | Cd | Cr | Cu | Ni | Pb | Zn |
| --- | --- | --- | --- | --- | --- | --- |
| Cd | 1 |  |  |  |  |  |
| Cr | 0.067 | 1 |  |  |  |  |
| Cu | 0.628** | 0.356 | 1 |  |  |  |
| Ni | 0.624** | 0.679** | 0.753** | 1 |  |  |
| Pb | -0.034 | 0.403* | 0.217 | 0.034 | 1 |  |
| Zn | 0.502* | 0.481* | 0.659** | 0.460* | 0.599** | 1 |

**Correlation is significant at the 0.01 level.

* Correlation is significant at the 0.05 level.


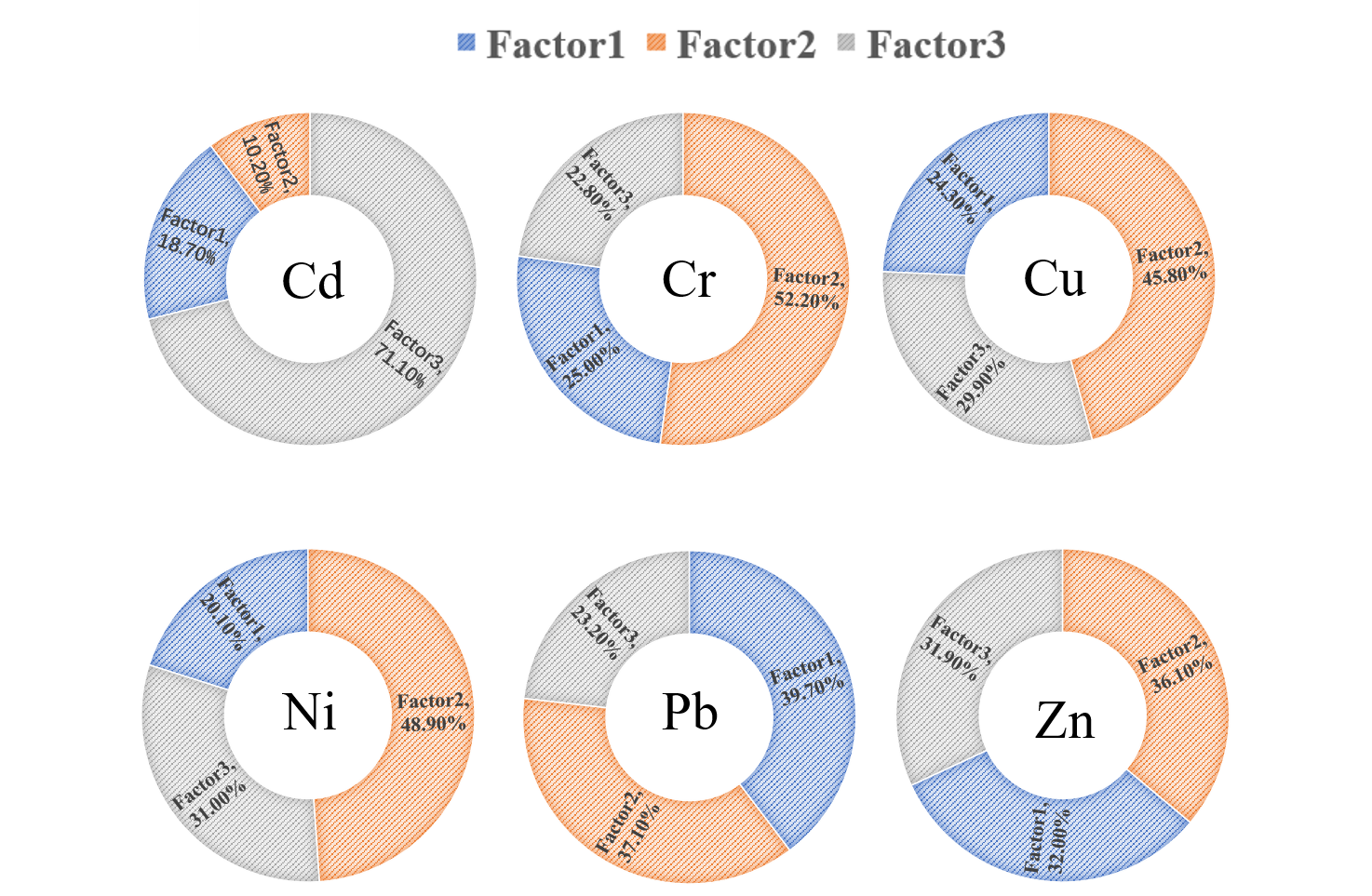


Figure S1. The contribution of each factor generated by the PMF model to the source of PTEs
